# A Rapid RPA-CRISPR/Cas12a Detection Method for Adulteration of Goat Milk Powder

**DOI:** 10.3390/foods12081569

**Published:** 2023-04-07

**Authors:** Shuqin Huang, Yan Liu, Xu Zhang, Zuoqi Gai, Hongtao Lei, Xing Shen

**Affiliations:** 1 Guangdong Provincial Key Laboratory of Food Quality and Safety/National-Local Joint Engineering Research Center for Machining and Safety of Livestock and Poultry Products, South China Agricultural University, Guangzhou 510642, China; 2 Guangzhou Editgene Co., Ltd., Guangzhou 510630, China; 3 Guangdong Laboratory for Lingnan Modern Agriculture, Guangzhou 510642, China

**Keywords:** goat milk powder, CRISPR/Cas12a, adulteration, rapid detection, food fraud

## Abstract

Because of the serious adulteration of goat milk, the rapid on-site detection of goat milk powder adulteration is needed. In this study, the CRISPR/Cas12a detection system combined with recombinase polymerase amplification (RPA) was employed to qualitatively detect the adulteration of goat milk powder with cattle-derived components. Specific primers and crRNA were designed and screened. After the optimization of RPA and the Cas system, the RPA-CRISPR/Cas12a detection method was established. The detection can complete the rapid identification of cattle-derived components in 45 min, without the assistant of large equipment. The absolute detectability of the RPA-CRISPR/Cas12a assay could reach 10^−2^ ng/μL for cattle genomic DNA, and 1% (*w/w*) for cattle milk powder, which is suitable to meet the testing requirements for on-site detection. In total, 55 commercial goat milk powder products were collected for blind testing. The results showed that 27.3% of the samples were adulterated with cattle ingredients, revealing a serious adulteration situation in goat milk powder market. The RPA-CRISPR/Cas12a assay established in this research exhibited its potential for practical use of on-site detection to detect cow milk powder in goat milk powder and can provide reliable technical reference for combating food fraud of adulteration of goat milk products.

## 1. Introduction

Since 1960s, goat milk has attracted people’s attention as high-value milk [1]. Its composition is similar to human milk, and it is rich in nutrients with low allergenicity [2]. In addition, goat milk contains active factors such as superoxide dismutase and DHA [3], which are beneficial to human health. In recent years, the market scale of goat milk has continued to rise, and a wide variety of goat milk products have been developed, among which goat milk powder is the product with the largest share in the market. However, due to the high nutritional and commercial value, goat milk products have a high risk of food fraud, especially for goat milk powder. Adulteration with cattle milk ingredients in the raw materials is the most common way because cattle milk has a similar composition, low cost, and is easy to access. In recent years, adulterations have been found in many sampling surveys of goat milk and its dairy products in recent years [4,5]. The adulteration not only infringes the interests of consumers, but also may cause food allergy. Therefore, to supervise this adulteration, various detection methods for goat milk products have been developed.

Several analytical techniques such as electrophoresis [6], spectroscopy [7], mass spectrometry [8], fingerprint analysis technology [9] were used for component analysis of goat milk products. These techniques depend on large and expensive instruments and complicated operations and are only suitable for laboratories. Immunoassay detects adulteration by identifying casein, which is abundant in milk, without complicated sample treatment. Among them, the lateral flow immunoassay [10,11] is the most convenient method. Although immunoassay is efficient and sensitive, it is not suitable for processed products because of protein denaturation.

Detection methods based on nucleic acid are another kind of major method and are considered to be mainstream methods in species identification with highly accurate and reliable results. At present, DNA-based methods developed for the identification of cattle-derived ingredients in goat milk products mainly include traditional PCR [12], DNA-based fluorometric methods [13], high resolution melting (HRM) [14], and real-time PCR [15,16]. Among all, real-time PCR is always regarded as the gold standard for the detection of animal-derived components. However, these methods also depend on precision instruments and professional technicians in the laboratory. The isothermal amplification technologies provided an easier way for on-site detection. LAMP (Loop-mediated isothermal amplification) has been used to detect adulteration of goat milk [17,18], but it is easy to produce false positive amplification for the complicated design of four to six primers. Moreover, after the process of drying and dehydration, the milk powder will further lose the nucleic acid, which hinders the detection methods development for milk powder, in spite of large-market occupancy of milk powder products. By now, the isothermal method, which is more convenient for the detection of milk powder, is still blank. The development of simple and rapid detection methods is urgently needed to meet the demand of on-site rapid adulteration detection of goat milk powder and other dairy products.

As a revolutionary tool for gene editing and regulation, CRISPR/Cas has later developed a rapid constant-temperature detection method [19]. This method has the advantages of simple operation, high sensitivity and specificity, and no reliance on precision instruments. At present, the CRISPR/Cas detection system has already been well applied in the fields of medical diagnosis, and it was also explored widely in the fields of food safety detection, which includes the detection of transgenic crops [20], meat adulteration [21], and food-borne pathogens [22,23]. However, the practical applications in real food products were still hysteretic, which may be attributed to the complexity of the food matrix. Especially in the field of food adulteration detection, there is a lack of on-site methods that own multiple advantages including being portable, fast, specific, and sensitive in one system. CRISPR/Cas is just such a promising method.

In this study, to meet the requirements of rapid detection on site, CRISPR/Cas12a combined with recombinase polymerase amplification (RPA) technology was used to establish a qualitative detection method for the adulteration of goat milk powder with cattle-derived components. After fully being evaluated with the specificity, sensitivity, and effectiveness, the developed method was applied to commercial samples for blind examination and proved to be suitable for practical use. The establishment of the RPA-CRISPR/Cas12a method can not only realize the on-site detection to detect cow milk powder in goat milk powder, but also provide technical reference for combating the species fraud of food.

## 2. Materials and Methods

### 2.1. Materials

All primers, ssDNAs, and CrRNA were synthesized by Genewiz Biotechnology (Suzhou, China). RPA assay kit was bought from TwistDW (Cambridge, UK). The Cas12a enzyme was provided by Kexin Biological Science and Technology Co., Ltd. (Beijing, China). RNase inhibitor was obtained from Hai Gene (Harbin, China). NEBuffer 2.1 was acquired from New England Biolabs Inc. (Ipswich, UK). DreamTaq DNA Polymerase was bought from Thermo Fisher Scientific (Waltham, MA, USA). The qPCR Probe Master Mix was bought from Vazyme Biotech Co., Ltd. (Nanjing, China). The cattle milk powder was acquired from Yili company (Hohhot, China). The standard goat milk powder was acquired from Hongxing Meiing Dairy Co., Ltd (Weinan, China), and soybean milk powder was acquired from Wuzhou Bingquan Industry Co., Ltd. (Wuzhou, China). A total of 55 whole goat milk powder products were collected from Chinese online shopping platforms including Taobao, Pinduoduo, and Tik Tok.

### 2.2. DNA Extraction

The sample DNA was extracted using an improved alkaline decomposition method [24]. The milk powder weighing 4 g was dissolved in 40 mL saturated saline. After centrifugation at 4 °C and 4500 rpm for 10 min, the supernatant was discarded, and the precipitate was resuspended with 1 mL of ddH_2_O and 200 μL of alkaline lysis buffer (0.5 mol/L NaOH, 0.1 mol/L Na_2_EDTA). The solution was incubated in boiling water for 5 min and centrifuged at 12,000 rpm for another 5 min. The supernatant was transferred to a new tube and mixed with 2.5 times the volume of absolute ethanol. The solution was placed at 4 °C for 30 min and then centrifuged at 4 °C and 8000 rpm for 10 min. After centrifugation, the supernatant was discarded, and the precipitate was resuspended with 1 mL of 70% ethanol and transferred to a new 1.5 mL tube, and then centrifuged at 4 °C and 12,000 rpm for 5 min. The supernatant was discarded again, and the precipitate was placed at room temperature for 5 min to dry and then resuspended with 50 μL ddH_2_O. The purified DNA was stored at −20 °C for further use.

### 2.3. Primer Design and RPA Amplification

With reference to the standard method “Technical Regulations for Authenticity Identification of Goat Milk” (NY/T 3050–2016), cattle mitochondrial DNA (NC_006853.1) was selected as the detection target in this study. RPA primers were designed according to the TwistDx^®^ instruction using Primer Premier 5.0 software and preliminarily screened by “Primer-BLAST” search “https://www.ncbi.nlm.nih.gov/tools/primer-blast/ (accessed on 19 April 2021)”. A total of 12 pairs of primers were synthesized and examined by an initial RPA system recommended by instruction manual. RPA reaction products were analyzed by agarose gel electrophoresis, and the primers resulting in a single amplified band with desired length were selected. Then the primers that were screened out were tested for their interspecies specificity by using the DNA of cattle, goat, sheep and soybean, respectively, extracted from cattle milk powder, goat milk powder, sheep milk powder, and soybean milk powder as templates. The best candidate primers were the primers with clear and bright electrophoresis target bands and no non-specific amplification except for cattle.

The best primer pair and cattle genomic DNA were used to optimize the RPA system including the concentration of primer, dNTPs, magnesium acetate (MgOAc), and the reaction time. The primer concentration was selected between 300 nM and 800 nM. The dNTPs concentration was set from 0.8 mM to 2.4 mM, and the MgOAc concentration was set from 7 mM to 24.5 mM. To meet the requirement of fast detection, the reaction time was optimized within half an hour. The reaction products were analyzed according to the fluorescence intensity provided by an initial CRISPR/Cas12a system, following the instruction manual of Cas12a enzyme. To lower the cost of the test, the volume of each reaction mixture used for the RPA reaction was reduced to 10 μL. 

### 2.4. Establishment of RPA-CRISPR/Cas12a Assay 

The crRNA probe was designed according to the target, and the sequence is UAAUUUCUACUAAGUGUAGAUCAUCUUUCAACUAAAAGUU. “BLAST” tool was used to verify the specificity of the crRNA probe designed by Cas-Designer “http://www.rgenome.net/cas-designer/ (accessed on 1 December 2021)”. Firstly, a series of concentrations of Cas12a enzyme (50 nM, 75 nM, 100 nM, 125 nM, and 150 nM) were selected. Based on the best concentration of Cas12a enzymes, different ratios of crRNA to the Cas12a enzyme (0.5:1, 1:1, 1.5:1, 2:1, and 2.5:1) were selected. Then the amount of RPA product used as a template was optimized.

In the RPA-CRISPR/Cas12a experimental operation, the reaction components of the RPA amplification system were mixed in a PCR tube, and MgOAc was finally added to the tube cover. Then, all the components were completely mixed by short centrifugation, and the reaction was started at 37 °C. After the RPA reaction was completed, 4 μL of RPA products were added to the CRISPR system, mixed evenly, and placed at 37 °C to start the reaction. When the cattle-derived components are identified, CRISPR system will produce a green fluorescence. The results can be observed with the naked eye by using a mini BluView Transilluminator (Eastwin Life Sciences, Inc., Beijing, China) under the wavelength of 470 nm, and photographed quickly with smartphone. The fluorescence intensity was collected immediately by QuantStudio 3 Real-Time PCR (Thermo Fisher Scientific, Waltham, MA, USA) at the same time. The no-treatment control (NTC) was set by using ddH_2_O as template. 

### 2.5. Specificity and Detectability of RPA-CRISPR/Cas12a

The DNA of cattle, goat, and soybean, which were extracted from respective kinds of milk powder, were used as templates at the same time to verify the specificity of the established RPA-CRISPR/Cas12a method for detection of cattle-derived ingredients. The absolute detectability of the method was tested upon diluted cattle genomic DNA with a concentration gradient from 10^−6^ ng/μL to 10^0^ ng/μL. At the same time, the limit of detection of the RPA-CRISPR/Cas12a assay was tested using a series of goat milk powder models adulterated with different proportions of cattle milk powder, and the proportions were set as 50%, 20%, 10%, 5%, 1%, 0.1% (*w*/*w*).

### 2.6. Real Sample Testing and Statistical Analysis 

In order to verify the application practicability of the established RPA-CRISPR/Cas12a method in commercially available products, 55 goat milk powder products of different brands were collected. DNA extraction from the samples followed the procedure described above. All the samples were analyzed by the RPA-CRISPR/Cas12a method and repeated three times for each testing. Green fluorescence generated by the sample indicates that the cattle-derived ingredients exist and the sample is positive. On the contrary, no green fluorescence indicates negative sample.

At the same time, the fluorescence intensity was read by Real-Time PCR and analyzed by SPSS 21.0 multivariate analysis software (IBM, Armonk, NY, USA). The experimental group and control group were compared by the unpaired two-tailed t-test, and the significant differences were indicated by *p*-value. *p* < 0.05 (*) was considered as statistically significant and indicated positive result here.

### 2.7. Method Validation 

In order to verify the accuracy of the established RPA-CRISPR/Cas12a method, all the 55 samples of goat milk powder products were analyzed by real-time PCR method at the same time, referring to the group standard “Qualitative detection method of bovine (domestic cattle, yak and buffalo) and sheep (goat and sheep) derived components in milk and dairy products (T/CNHFA 002-2022, China)”. When the mean CT ≤ 30, the sample was judged as positive. When mean CT ≥ 35, it was judged as negative. When 30 < mean CT < 35, the test was repeated, and if the result Ct < 35, it was judged as positive. Each sample was amplified with targeted gene and reference gene at the same time. A positive control, a negative control, and a blank control using cattle DNA, goat DNA and ddH_2_O as template, respectively, were incorporated in the experiment. The experiment is effective when the reference gene detection and positive control are positive, and the negative control and blank control are negative. 

## 3. Results and Discussion

### 3.1. Primers Screening and RPA Optimization

**Figure 1:**
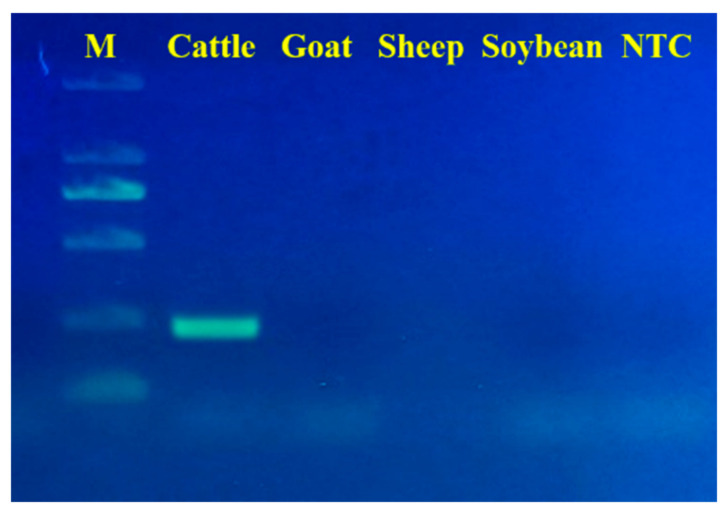
Amplification result based on the best RPA primers for cattle genomic DNA. NTC: nontarget control.

After comparing and analyzing the homology of mtDNA sequences within and between species, 12 pairs of cattle-specific RPA primers were designed and selected from cattle mitochondrial gene (NC_006853.1, nt 8133~8371). The specificity of the primers was examined among different species of cattle, goat, sheep, and soybean. The amplification result of the best pair of primers showed that the only band was amplified from cattle DNA, and none from others (Figure 1). The sequence of the final selected primers is 5′ GGGTTACGAGAGGGAGACCTAAAATTACAG-3′ for forward primer, and 5′-TTGGGAATAGTACGATGCCGCAACTAGACA-3′ for reverse primer.

**Figure 2:**
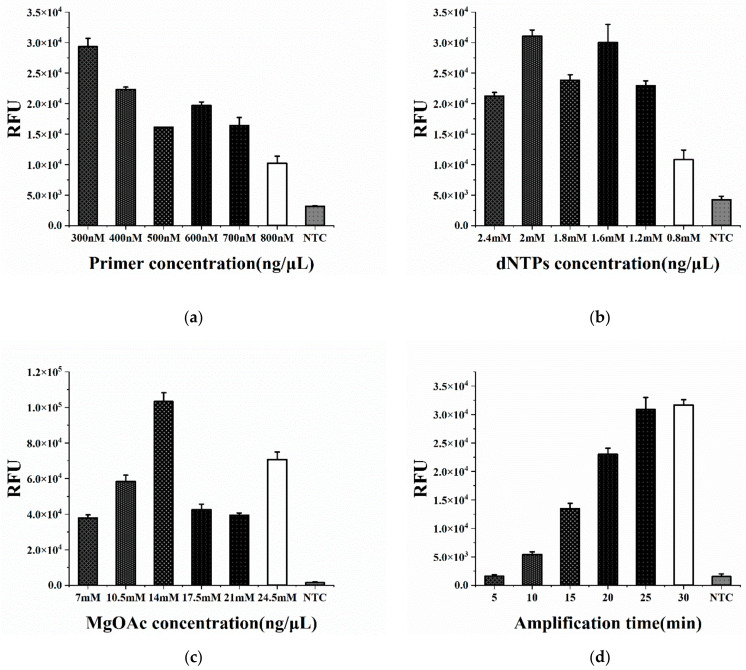
**Condition** optimization of RPA amplification system. (**a**) Primer concentration. (**b**) dNTPs concen-tration. (**c**) MgOAc concentration. (**d**) Amplification time. NTC: nontarget control.

In order to improve the amplification efficiency, the amplification conditions were optimized. Primer concentration is an important factor affecting RPA amplification. High primer concentration will lead to mismatches and dimer formation, while low primer concentration will lead to insufficient amplification products, but it is beneficial to long amplicon and improves real-time resolution. In this study, the fluorescence value showed an overall downward trend with the increase in the primer concentration, and the maximum amplification efficiency was obtained when the primer concentration was 300 nM (Figure 2a). dNTPs provide the raw materials for amplification. With the increase in the dNTPs amount, the fluorescence intensity first increases and then decreases, and the optimal concentration was 2 mM (Figure 2b). MgOAc provides energy for amplification. The results showed that different concentrations of MgOAc did not cause regular fluorescence changes. According to the highest fluorescence value, the optimal MgOAc concentration was 14 mM (Figure 2c). As the amplification reaction proceeded, the amount of amplification products continually accumulated. When the amplification time is 25 min, the fluorescence is close to saturation, which indicates that the amplification can be completed within 25 min under the optimal reaction conditions (Figure 2d). The final reaction mixture contained 10 μM forward primer, 10 μM reverse primer, 40 mM dNTPs, 280 mM MgOAc, 1.95 μL of the DNA template, 5 μL of the 2× reaction buffer, 1 μL of 10× Basic E-mix, and 0.5 μL of 20× Core Reaction Mix. The reaction was carried out at the constant temperature of 37 °C for 25 min. 

### 3.2. Establishment of RPA-CRISPR/Cas12a Method

**Figure 3:**
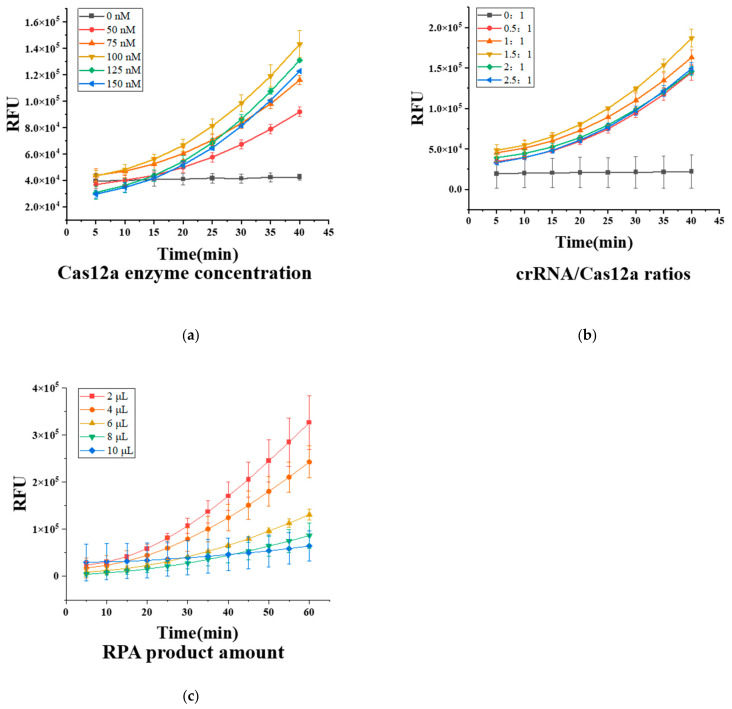
Optimization of RPA-CRISPR/Cas12a assay. (**a**) Cas12a enzyme concentration. (**b**) crR-NA/Cas12a ratios. (**c**) RPA product amount.

In the CRISPR/Cas12a system, Cas12a, crRNA and target DNA form a ternary complex to activate the reaction. The concentration of Cas12a directly affects the reaction rate. With the increase in the Cas12a enzyme concentration, the reaction efficiency first increased and then decreased, and reached the highest when the Cas12a enzyme concentration was 100 nM (Figure 3a). Because the amount of crRNA should match Cas12a, further optimization was achieved by changing the ratios of crRNA to the Cas12a enzyme. With the proportion of crRNA increased, the fluorescence intensity also showed a trend of rise at first, and then declined. Therefore, the optimal ratios of crRNA to the Cas12a enzyme chose 1.5:1 (Figure 3b). At last, the amount of the RPA product used was also optimized. Although more RPA product could provide more DNA target for the Cas enzyme, the result in this study exhibited a continuous decrease in the reaction efficiency while more RPA product was used (Figure 3c). The reason may be that the complex RPA reaction system interferes with the activity of the Cas enzyme. Considering that in actual operation, too few DNA targets would lead to unstable experimental results, the amount of the RPA product used is finally set at 4 μL. The optimal Cas12a enzyme reaction system is consisted of 1 μM Cas12a, 1 μM CrRNA, 10 μM reporter ssDNA, 0.8 U/μL RNase inhibitor, 2.5 μL of 10 × NEBuffer 2.1, 4 μL of template DNA, and complemented to 20 μL with ddH_2_O. The optimal CRISPR/Cas12a system can produce enough visible green fluorescence under the blue light within 20 min. 

Combined with the RPA steps, the whole RPA-CRISPR/Cas12a detection can be completed in 45 min under a constant temperature of 37 °C, without the assistance of any large equipment. It is suitable for the on-site detection of goat milk powder due to the mild detection conditions, simple operation, and portable devices.

### 3.3. The Specificity and Sensitivity of RPA-CRISPR/Cas12a Method

**Figure 4:**
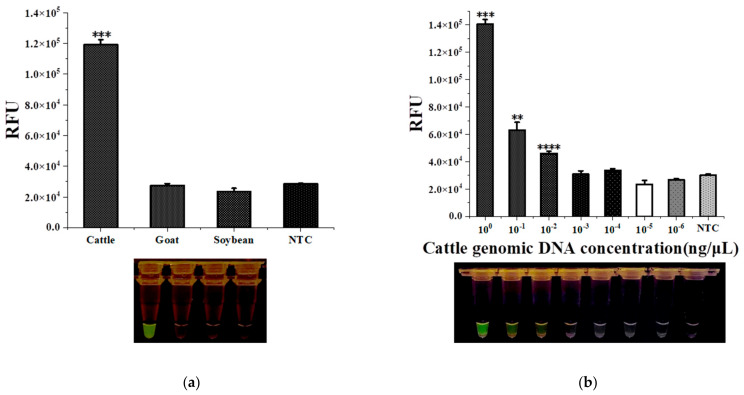

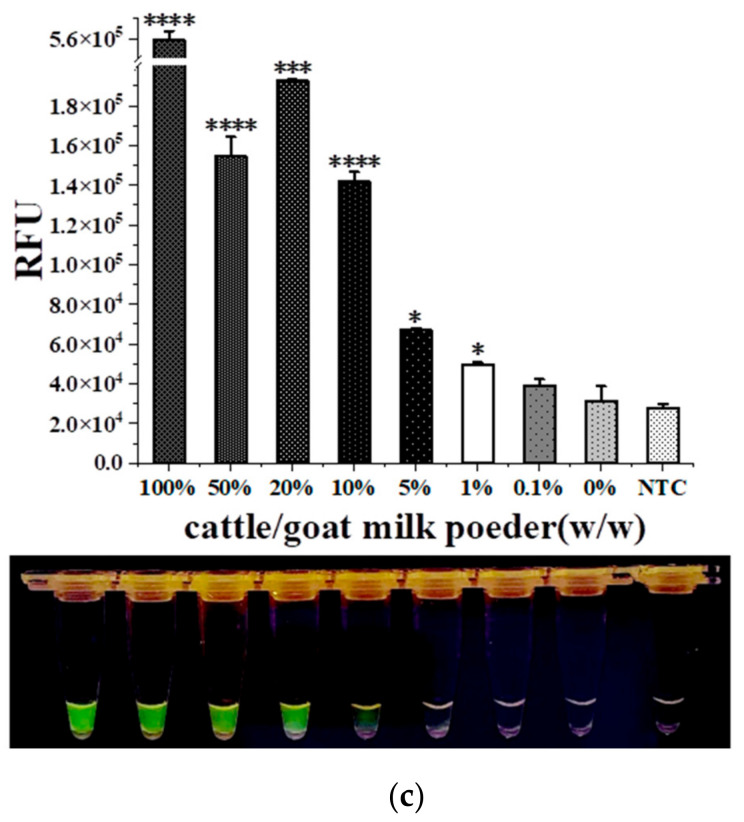
Specificity and detectability of RPA-CRISPR/Cas12a. (**a**) The specificity of RPA-CRISPR/Cas12a. (**b**) The absolute detectability of RPA-CRISPR/Cas12a. (**c**) The detection limit of adulteration ratio. NTC: nontarget control (ddH_2_O) (* *p* < 0.05, ** *p* < 0.01, *** *p* < 0.001, **** *p* < 0.0001).

The DNA of cattle, goat, and soybean were used as templates to verify the specificity of the established RPA-CRISPR/Cas12a assay. The experimental results showed that only cattle DNA could activate the Cas12a enzyme and emit a visible fluorescence (Figure 4a), while the other species showed no significant difference from the blank control. It proved that the established RPA-CRISPR/Cas12a method has good specificity for the recognition of cattle-derived components. 

The detectability of the established RPA-CRISPR/Cas12a assay was identified from two levels. On one hand, the absolute sensitivity was tested at the genomic DNA level. The cattle genomic DNA from 10^−2^ to 10^0^ ng produced a visible green fluorescence that could be distinguished by the naked eye (Figure 4b). The statistical analysis result was the same with that of the naked eye. A significant fluorescence signal that was different from the blank control (*p* < 0.01) was obtained when the cattle genomic DNA was ≥ 10^−2^ ng/μL. This indicated that the absolute detectability of the established RPA-CRISPR/Cas12a assay could reach to 10^−2^ ng/μL for cattle genomic DNA. Compared with other methods that have been reported to detect the adulteration in goat milk, such as the QPCR method with the detection limit of 0.01 ng/μL cattle DNA [25], and LAMP method with the detection limit of 0.05 ng/µL cattle DNA [18], this method has equal sensitivity. However, it is harder to detect the adulteration of milk powder than raw milk, due to the difficulties of DNA collection from milk powder. On the other hand, to meet the requirement of practical application, the limit of detection was also determined by a series of goat milk powder models adulterated with different proportions of cattle milk powder. The results (Figure 4c) showed that obvious fluorescence could be read by the naked eye until 5% (*w*/*w*) adulteration proportion. Through data analysis, when the adulteration proportion ≥ 1%, the fluorescence value was significantly different from NTC, and the detection limit can reach 1% (*w*/*w*). In general, the adulteration ratio will not be lower than 1% in food fraud, for the sake of pursuing economic benefit. A LAMP method has been developed to detect goat milk, and the detection limit was 1% milk [18]. By contrast, this method fills the blank of rapid detection methods of goat milk powder and has a good detection ability for on-site detection of milk powder. The detection limit can well meet the requirement of adulteration detection of goat milk powder in market.

### 3.4. Real Sample Testing

**Figure 5:**
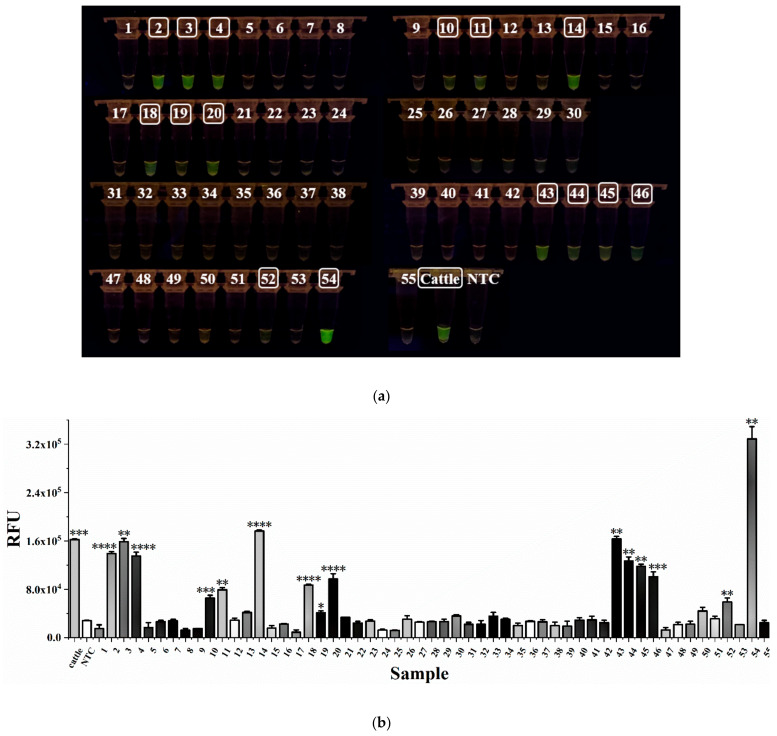
The results of 55 goat milk powder products tested by RPA-CRISPR/Cas12a assay. (**a**) The result of naked-eye observation. The white boxes show that 15 samples were found to contain cattle-derived ingredients. (**b**) The result of data analysis. The unpaired two-tailed Student’s t-test was used to analyze the statistical significance of the sample fluorescence value and NTC (* *p* < 0.05, ** *p* < 0.01, *** *p* < 0.001, **** *p* < 0.0001). NTC: negative control; Cattle: positive control.

In total, 55 goat milk powder products were collected from the three hottest online shopping platforms in China, for the purpose of verifying the feasibility of the established RPA-CRISPR/Cas12a rapid detection method and making a small-scale investigation of the adulteration situation of the current goat milk powder market at the same time. The results showed that 15 samples of goat milk powder produced an obvious green fluorescence that can be observed by the naked eye (Figure 5a). The results of statistical analysis are totally consistent with that of naked-eye observation (Figure 5b). In summary, among the 55 goat milk powder products sold in the online markets, 15 samples were found to be adulterated with cattle-derived components, which is a 27.3% adulteration rate. In the other two studies, in 2019 and 2020, about ten real samples of goat milk powder were tested, and 2–3 samples in 10 were identified as adulterated with cattle-derived ingredient. The detection results tend to be consistent with this study [26,27]. The small-scale investigation in this study collected more commercial products and revealed that the adulteration of goat milk powder products is still serious at present.

### 3.5. Method Validation

The testing results of 55 goat milk powder products were confirmed by a qPCR method from the group standard (T/CNHFA 002-2022) for method validation. The qPCR results showed a total of 15 samples were positive for cattle-derived components (Figure S1 and Table S1). Although the tests were repeated many times, No. 47 and No. 50 samples cannot be detected in the control group, which used the mammalian GH gene as reference gene. Therefore, the tests for the two samples were invalid. Then we found that the prices of the two samples are extremely low, and that there is a possibility of adulteration by completely replacing goat milk powder with non-animal ingredients. Except for this, all the positive samples were consistent with the results of the positive samples detected by the RPA-CRISPR/Cas12a assay, which indicated that the established RPA-CRISPR/Cas12a method has good precision and can be applied to the identification of cattle-derived components in commercial goat milk powder products.

## 4. Conclusions

In this study, a rapid RPA-CRISPR/Cas12a qualitative detection method was established for the adulteration of goat milk powder with cattle-derived components. The entire detection reaction can be carried out at a constant 37 °C, and the result can be easily judged by the naked eye under a portable blue light transilluminator. The detection limit of this method can well meet the requirements of the food monitoring while maintaining high specificity. A small-scale investigation of 55 commercial goat milk powder products revealed the serious situation of adulteration nowadays. Therefore, the RPA-CRISPR/Cas12a assay established in this research, which is very suitable for the rapid on-site detection for the identification of cattle-derived ingredients in goat milk powder, can provide reliable technical reference for combating the food fraud of adulterating goat milk products.

## Data Availability

Data are contained within the article (or Supplementary Material).

## Supplementary Materials

The following supporting information can be downloaded at: https://www.mdpi.com/article/10.3390/foods12081569/s1, Table S1: Comparison of RPA-CRISPR/Cas12a and qPCR method for Real sample testing; Figure S1: The results of cattle-derived ingredient in 55 goat milk powder products tested by qPCR method.
