# Supplementary material for: A Rapid RPA-CRISPR/Cas12a Detection Method for Adulteration of Goat Milk Powder
Source: Foods. 2023 Apr 7;12(8):1569. doi: 10.3390/foods12081569 (PMC10137891; doi:10.3390/foods12081569)
Supplement: Supplementary file 1 [file foods-12-01569-s001.zip › foods-2253963-Supplementary File.pdf]

Table S1. Comparison of RPA-CRISPR/Cas12a and qPCR method for Real sample testing.

ID	Real-time PCR assay			RPA-CRISPR/Cas12a assay	ID	Real-time PCR assay			RPA-CRISPR/Cas12a assay
	Target	Mean Ct	Result			Target	Mean Ct	Result	
1	Cattle	*[a]	−[b]	−	19	Cattle	34.22	+[c]	+
	Control	27.58				Control	34.87		
2	Cattle	29.71	+	+	20	Cattle	28.63	+	+
	Control	25.08				Control	28.47		
3	Cattle	29.27	+	+	21	Cattle	*	−	−
	Control	26.99				Control	29.70		
4	Cattle	30.68	+	+	22	Cattle	*	−	−
	Control	27.01				Control	28.92		
5	Cattle	*	−	−	23	Cattle	*	−	−
	Control	26.08				Control	27.03		
6	Cattle	*	−	−	24	Cattle	*	−	−
	Control	24.72				Control	29.26		
7	Cattle	*	−	−	25	Cattle	*	−	−
	Control	32.28				Control	23.44		
8	Cattle	*	−	−	26	Cattle	*	−	−
	Control	27.75				Control	28.96		
9	Cattle	*	−	−	27	Cattle	*	−	−
	Control	27.03				Control	27.66		
10	Cattle	32.03	+	+	28	Cattle	*	−	−
	Control	26.37				Control	29.38		
11	Cattle	30.86	+	+	29	Cattle	*	−	−
	Control	25.81				Control	27.53		
12	Cattle	*	−	−	30	Cattle	*	−	−
	Control	33.80				Control	36.43		
13	Cattle	*	−	−	31	Cattle	*	−	−
	Control	25.35				Control	29.76		
14	Cattle	29.44	+	+	32	Cattle	*	−	−
	Control	26.57				Control	28.69		
15	Cattle	*	−	−	33	Cattle	*	−	−
	Control	31.69				Control	32.93		
16	Cattle	*	−	−	34	Cattle	*	−	−
	Control	23.47				Control	29.98		
17	Cattle	*	−	−	35	Cattle	*	−	−
	Control	28.94				Control	31.67		
18	Cattle	29.23	+	+	36	Cattle	*	−	−
	Control	32.04				Control	27.42		

Continue

37	Cattle	*	−	−	48	Cattle	*	−	−
	Control	27.99				Control	30.63		
38	Cattle	*	−	−	49	Cattle	*	−	−
	Control	32.77				Control	30.22		
39	Cattle	*	−	−	50	Cattle	*	−	−
	Control	26.97				Control	*		
40	Cattle	*	−	−	51	Cattle	*	−	−
	Control	30.55				Control	30.86		
41	Cattle	*	−	−	52	Cattle	34.85	+	+
	Control	23.29				Control	35.05		
42	Cattle	*	−	−	53	Cattle	*	−	−
	Control	28.60				Control	31.60		
43	Cattle	33.64	+	+	54	Cattle	30.20	+	+
	Control	34.47				Control	27.65		
44	Cattle	34.63	+	+	55	Cattle	*	−	−
	Control	34.42				Control	28.35		
45	Cattle	33.01	+	+	55	Cattle	*	−	−
	Control	33.20				Control	28.35		
46	Cattle	34.69	+	+	Cattle	Cattle	32.09	+	+
	Control	35.60				Control	32.28		
47	Cattle	*	−	−	NTC	Cattle	*	−	−
	Control	*				Control	*		

a (*): Mean Ct > 40.

b (—): Negative for cattle-derived ingredient.

c (+): Positive for cattle-derived ingredient.

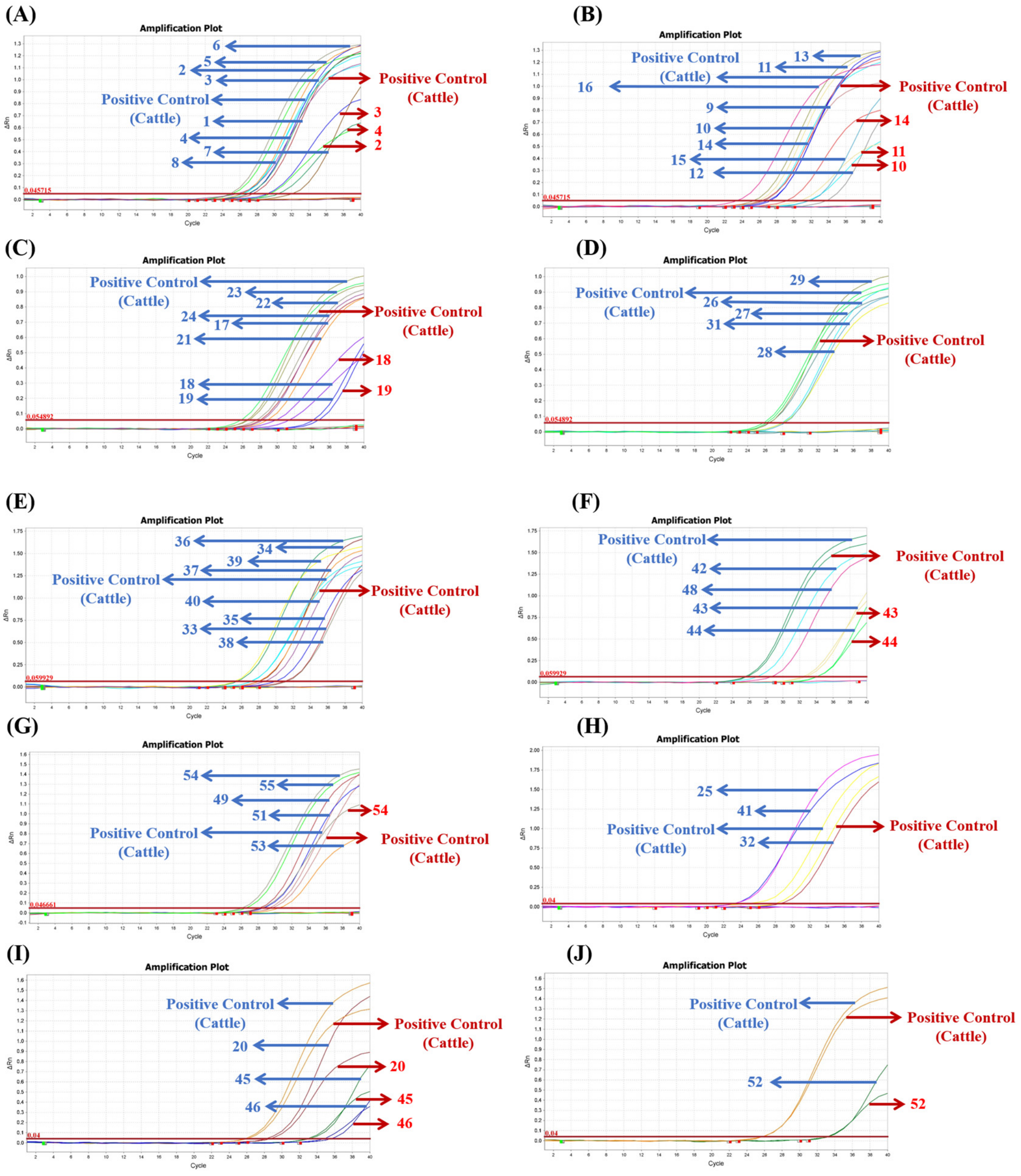

Figure S1. The results of cattle-derived ingredient in 55 goat milk powder products tested by qPCR method.
